# The role of renin angiotensin system antagonists in the prevention of doxorubicin and trastuzumab induced cardiotoxicity

**DOI:** 10.1186/s12947-015-0011-x

**Published:** 2015-04-03

**Authors:** Gauri Akolkar, Navdeep Bhullar, Hilary Bews, Bilal Shaikh, Sheena Premecz, Kimberly-Ann Bordun, David YC Cheung, Vineet Goyal, Anita K Sharma, Philip Garber, Pawan K Singal, Davinder S Jassal

**Affiliations:** Institute of Cardiovascular Sciences, St. Boniface Research Centre, University of Manitoba, Winnipeg, Manitoba Canada; Section of Cardiology, Department of Internal Medicine, University of Manitoba, Winnipeg, Manitoba Canada; Section of Oncology, Department of Internal Medicine, University of Manitoba, Winnipeg, Manitoba Canada; Department of Radiology, St. Boniface General Hospital, University of Manitoba, Winnipeg, Manitoba Canada; Associate Professor of Medicine, Radiology, and Physiology, Section of Cardiology, Department of Internal Medicine, College of Medicine, Faculty of Health Sciences, Rm Y3531, Bergen Cardiac Care Centre, St. Boniface General Hospital, 409 Tache Avenue, Winnipeg, Manitoba R2H 2A6 Canada

**Keywords:** Cardio-Oncology, Doxorubicin, Trastuzumab, RAS antagonists, Murine echocardiography

## Abstract

**Background:**

Cardio-Oncology is an evolving discipline that focuses on the management of cancer patients who develop cardiovascular complications as a result of their treatment. Although the current combination of surgical resection, radiation, and chemotherapy may lead to a cure in cancer patients, the administration of anti-cancer drugs, in particular Doxorubicin (DOX) and Trastuzumab (TRZ), is associated with an increased risk of cardiotoxicity. Little is known on the potential cardioprotective role of renin angiotensin system (RAS) antagonists in the prevention of DOX+TRZ mediated cardiotoxicity.

**Objective:**

The aim of the study was to determine whether RAS antagonists would be useful in attenuating DOX+TRZ induced cardiotoxicity.

**Methods:**

A total of 240 C57Bl/6 mice were randomized to prophylactic treatment with placebo, Aliskiren, Perindopril, or Valsartan for a total of 13 weeks. Within each arm, mice received treatment with either DOX, TRZ, or the combination of both drugs. Serial murine echocardiography was performed weekly to characterize the degree of cardiovascular remodeling within each group.

**Results:**

In wild-type (WT) mice treated with DOX+TRZ, LV end diastolic internal diameter (LVID) increased from 3.1 ± 0.2 mm at baseline to 4.6 ± 0.3 mm at week 13 (p < 0.05) and the LV fractional shortening (FS) decreased from 52 ± 2% at baseline to 26 ± 2% at week 13 (p < 0.05). Prophylactic treatment with Aliskiren, Perindopril, or Valsartan attenuated the degree of LV cavity dilatation with LVID dimensions of 3.9 ± 0.2 mm, 4.1 ± 0.2 mm, and 4.2 ± 0.1 mm at week 13, respectively (p < 0.05). Similarly, prophylactic treatment with Aliskiren, Perindopril, or Valsartan was partially cardioprotective with FS of 40 ± 1%, 32 ± 1%, and 33 ± 2% at week 13, respectively (p < 0.05). As compared to WT mice receiving DOX+TRZ, prophylactic treatment with RAS inhibition was also associated with improved survival, corroborating the echocardiographic findings.

**Conclusion:**

The cardiotoxic effects of DOX+TRZ were partially attenuated by the prophylactic administration of RAS antagonists in a chronic murine model of chemotherapy induced cardiac dysfunction.

## Introduction

As the second leading cause of cancer related deaths in women, breast cancer constitutes a major health concern. In Canada alone, approximately 22,000 women are diagnosed with breast cancer each year and over 5,000 die from the disease [1,2]. Anthracyclines, including Doxorubicin (DOX), are a class of chemotherapeutic agents with demonstrated efficacy in the breast cancer setting [3-6]. The clinical utility of DOX is limited, however, by its inherent cardiotoxicity. The average incidence of DOX induced heart failure is 5% at a cumulative dose of 400 mg/m^2^ and increases to 25% with cumulative doses above 550 mg/m^2^ [3-6]. In addition to DOX, Trastuzumab (TRZ), a monoclonal antibody against the extracellular domain of the human epidermal growth factor receptor 2 protein (HER2), is used in both the adjuvant and metastatic settings of HER2 positive breast cancer [7-12]. The addition of TRZ to adjuvant chemotherapy in patients with HER2 positive breast cancer was unequivocally beneficial, facilitating a 50% decrease in the risk of cancer relapse and a 33% decrease in mortality [7-12]. Despite its widespread adoption, however, it is now recognized that TRZ potentiates the cardiotoxic side effects of DOX. Although clinical trials have reported the risk of developing LV systolic dysfunction following DOX+TRZ treatment to be as great as 10% [9-11], recent real world studies have indicated that nearly 1 in 4 women will develop a drug induced cardiomyopathy, demonstrating the clinical significance of this disease [13-17].

Among the potential mechanisms for DOX+TRZ mediated cardiotoxicity, activation of the renin-angiotensin system (RAS) has gained recent attention. In an animal model of chemotherapy induced cardiomyopathy, chronic DOX administration (1 mg/kg biweekly, for 8 weeks) was associated with a 2-fold increase in plasma renin activity, the enzyme responsible for initiating the RAS [18]. In a separate study, cardiac neurohumoral activation, signified by increased expression of pro-atrial naturetic peptide (ANP) mRNA, was observed in rabbits treated with 2 mg/kg DOX [19]. As RAS activation was shown to trigger ANP expression, these findings suggested that cardiac neurohumoral activation was mediated through an increase in the RAS [20]. Finally, in a chronic rat model, a cumulative dose of 420 mg/kg DOX facilitated up-regulation of the angiotensin II type 1 receptor (AT1_R_) [21]. Additional studies have demonstrated the downstream cardiotoxic effects of AT1_R_ signalling, including stimulation of NADPH oxidase to generate oxidative stress, and activation of mitogen-activated protein kinases (MAPKs), responsible for cellular growth, inflammation, hypertrophy, and apoptosis [22,23].

RAS involvement in the pathophysiology of DOX+TRZ mediated cardiac dysfunction has raised the question as to whether the prophylactic use of RAS antagonists could potentially mitigate these cardiotoxic effects. Previous basic science studies have demonstrated that the prophylactic administration of angiotensin converting enzyme inhibition (ACEI), including Captopril, Enalapril, and Lisinopril, was partially cardioprotective in both acute and chronic animal models of DOX induced cardiomyopathy [19,24,25]. In a rabbit model of DOX mediated cardiomyopathy, 1 mg/kg/day oral Lisinopril for a total of 10 weeks attenuated cardiomyocyte loss and ANP mRNA expression, in comparison to rabbits receiving DOX alone [19]. Furthermore, intragastric administration of Captopril (10 mg/kg) or Enalapril (2 mg/kg) for 7 days resulted in a decline in lipid peroxidation, and enzymatic indicators of acute cardiac toxicity in a rat model of DOX induced cardiomyopathy [24]. Little is known, however, on the utility of antagonizing the RAS pathway in a hierarchical fashion for the prevention of combined DOX+TRZ mediated cardiotoxicity.

The objective of the current study was to determine whether inhibition of the RAS pathway at three distinct levels, including direct renin inhibition (DRI; Aliskiren), angiotensin converting enzyme inhibition (ACEI; Perindopril), and angiotensin receptor blockade (ARB; Valsartan) can prevent cardiac dysfunction in a chronic *in vivo* model of DOX+TRZ mediated cardiotoxicity.

## Methods

### Experimental animals

Animal procedures were conducted in accordance with guidelines published by the Canadian Council on Animal Care. All procedures, including drug administration and longitudinal echocardiographic studies, were approved by the Animal Protocol Review Committee at the University of Manitoba.

Adult male C57Bl/6 mice, weighing 20–25 g, were obtained from Jackson Laboratories (MA, US). The animals were housed under controlled environmental conditions, including temperature, humidity, and lighting. Standard laboratory chow and water were provided ad libitum.

### Experimental protocol

**Figure 1:**
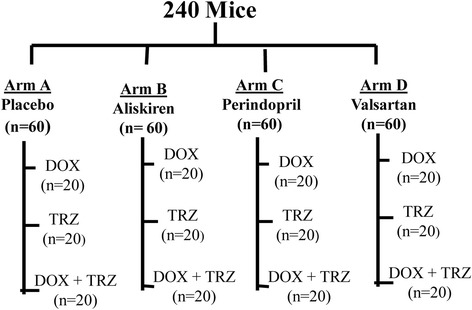
**A total of 240 C57Bl/6 mice were randomized to one of the following prophylactic treatment arms: (A) placebo (saline; n = 60); (B) Aliskiren (50 mg/kg; n = 60); (C) Perindopril (3 mg/kg; n = 60); or (D) Valsartan (10 mg/kg; n = 60).** RAS antagonists were administered orally on a daily basis for the entire study period of 13 weeks. Furthermore, mice from each prophylactic treatment arm were randomized to one of the following chemotherapeutic regimens: (i) TRZ (4 mg/kg weekly, intraperitoneal (i.p.); n = 20); (ii) DOX (4 mg/kg weekly, i.p.; n = 20); or (iii) DOX+TRZ (n = 20).

**Figure 2:**
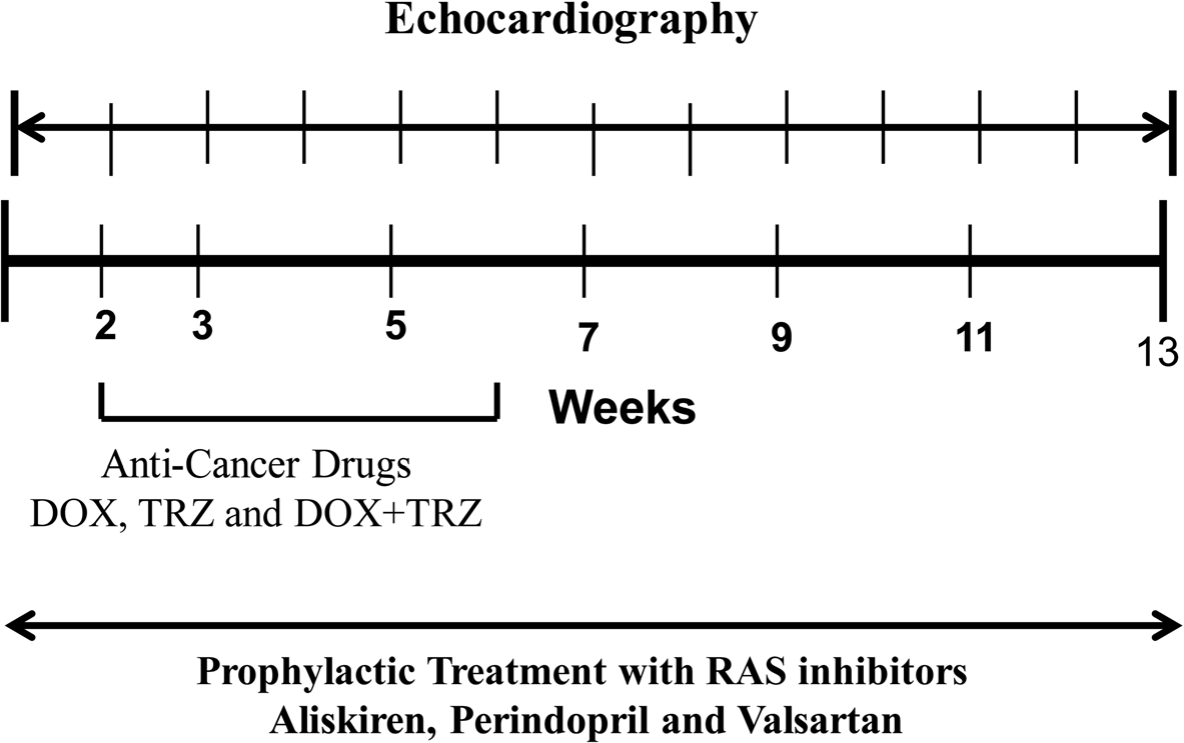
**Timeline for drugs administered to mice in each group.** Mice received prophylactic treatment with Aliskiren (50 mg/kg), Perindopril (3 mg/kg) or Valsartan (10 mg/kg) in drinking water daily. Prophylactic treatment was started 2 weeks prior to chemotherapy with DOX, TRZ and DOX + TRZ and was continued for 13 weeks. DOX (4 mg/kg), TRZ (4 mg/kg) and DOX + TRZ (4 mg/kg) was administered by weekly intraperitoneal injection for a total of 5 weeks. Cardiac function was monitored by echocardiography on a weekly basis for 13 weeks.

A total of 240 C57Bl/6 male mice were randomized to one of the following prophylactic treatment arms: (A) placebo (saline; n = 60); (B) Aliskiren (50 mg/kg; n = 60); (C) Perindopril (3 mg/kg; n = 60); or (D) Valsartan (10 mg/kg; n = 60) (Figure 1). RAS antagonists were administered orally by gavage on a daily basis for the entire study period of 13 weeks. Furthermore, mice from each prophylactic treatment arm were randomized to one of the following chemotherapeutic regimens: (i) TRZ (4 mg/kg weekly, intraperitoneal (i.p.); n = 20); (ii) DOX (4 mg/kg weekly, i.p.; n = 20); or (iii) DOX+TRZ (n = 20) (Figure 1). TRZ, DOX, or DOX+TRZ injections were initiated at week 2, following 2 weeks of prophylactic treatment with a RAS antagonist or placebo, and continued for 5 weeks (Figure 2). The cumulative doses of DOX or TRZ achieved were the minimum concentration to induce a chemotherapy mediated cardiomyopathy, as previously validated by our group and others [26-28]. Cardiac function was evaluated over the course of the study via serial murine echocardiography. Mice were imaged at baseline and weekly until euthanization at week 13.

### Murine echocardiography

*In vivo* cardiac function was evaluated by serial murine echocardiography, as previously described [27]. Awake mice were imaged at baseline and weekly thereafter, for the duration of the 13-week study (Figure 2). Images were captured in M-mode and the 2D parasternal short axis view, using a 13-MHz linear array ultrasound probe (Vivid 7, GE Medical Systems, Milwaukee, WI, USA). M-mode recordings were used to evaluate indices of cardiac dimension and function: LV end diastolic internal diameter (LVID), LV end systolic internal diameter (LVIS), interventricular septal thickness (IVS), posterior wall thickness (PWT), LV fractional shortening (LVFS), and LV ejection fraction (LVEF). Echocardiographic images were processed offline using EchoPAC v110.0.0 PC software (Vivid 7, GE Medical Systems, Milwaukee, WI, US).

A total of 40 mice, from various treatment groups, were randomly selected in order to assess the variability associated with echocardiographic measurements of LV cavity dimensions and function. To determine intra-observer variability, repeat LVID and LVFS measurements were made by a single observer (DJ), two weeks apart. Inter-observer variability was established by comparing LVID and LVFS values calculated by two independent observers (NB and DJ). Intra- and inter-observer variability were reported as the absolute value of the difference between the two observations divided by the mean.

### Statistical analysis

Statistical analysis was conducted using SPSS 15.0 and GraphPad Prism 5, with echocardiographic data expressed as a mean ± SD. An analysis of variance approach for repeated measurements comparison of echocardiographic parameters over time was used. One-way ANOVA and post-hoc Dunnett’s tests were performed when necessary. A P value < 0.05 was considered statistically significant.

## Results

### Survival

**Figure 3:**
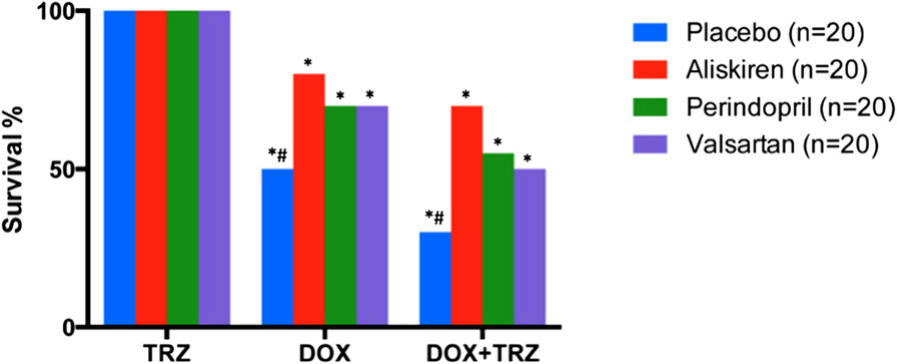
**Survival probability at the end of 13 weeks for C57Bl/6 mice receiving prophylactic treatment with RAS inhibitors Aliskiren, Perindopril or Valsartan and then treated with anti-cancer drugs TRZ, DOX or the combination of DOX+TRZ.** *p < 0.05 significant as compared to TRZ. ^#^p < 0.05 comparing placebo to either Aliskiren, Perindopril, or Valsartan.

The overall survival of C57Bl/6 mice administered TRZ, DOX, or DOX+TRZ, with or without RAS inhibitor prophylaxis is represented in Figure 3. In mice treated with TRZ alone, the survival rate was 100%. The administration of either DOX alone or the combination of DOX+TRZ resulted in survival rates to be significantly reduced to 50% and 30%, respectively, by the end of the study. Conversely, prophylactic administration of a RAS inhibitor attenuated the increased mortality rates observed with both DOX and DOX+TRZ therapy. For mice in the DOX treatment arm, overall survival was significantly increased when pretreated with Aliskiren, Perindopril, or Valsartan (p < 0.05). Similarly, overall survival following DOX+TRZ treatment was significantly increased by the prophylactic administration of one of the RAS antagonists (Figure 3).

### Echocardiographic indices

**Figure 4:**
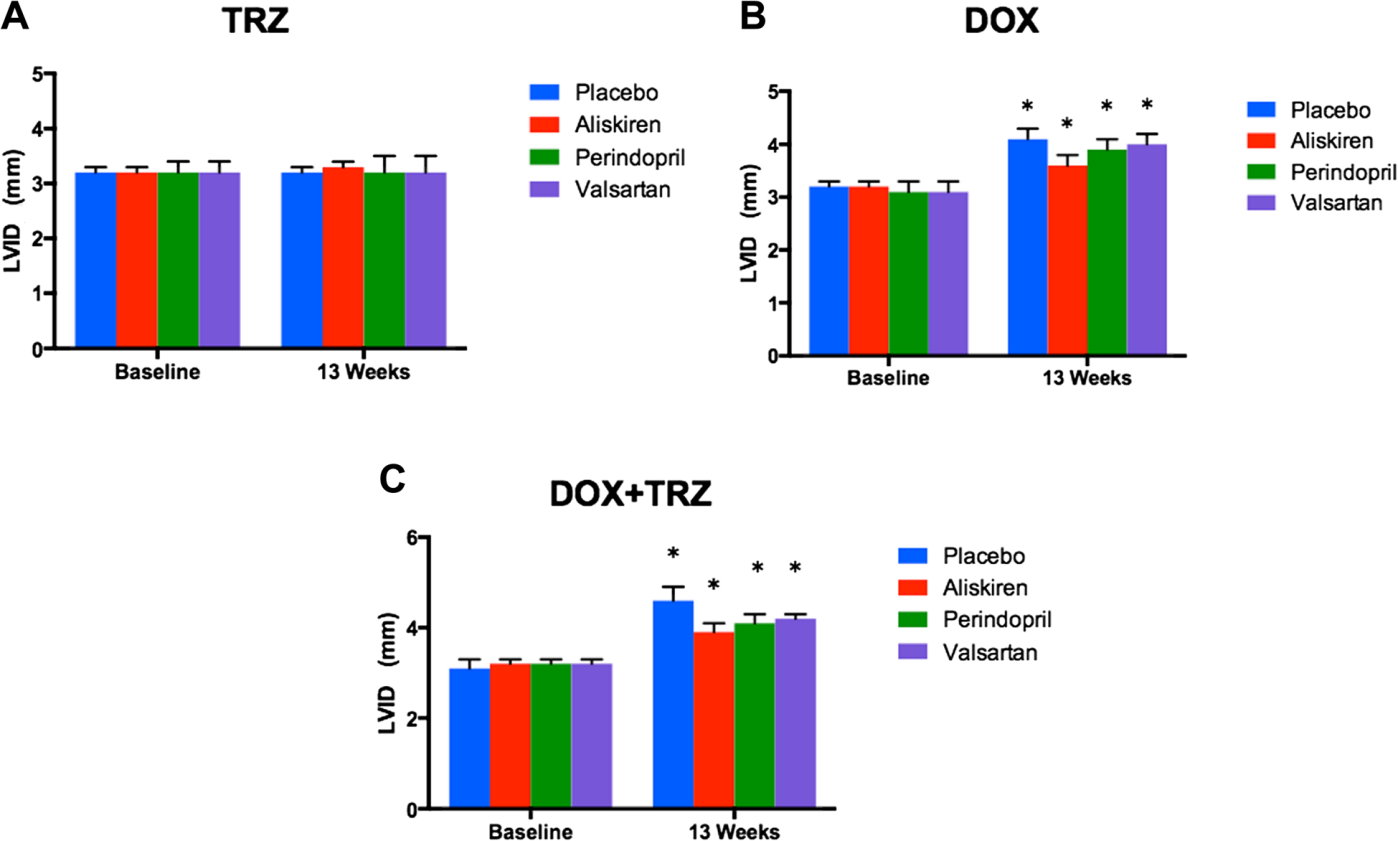
**LVID (mm) in C57Bl/6 mice receiving prophylactic treatment with Aliskiren, Perindopril, Valsartan or placebo prior to receiving one of the following anti-cancer drugs: A) TRZ; B) DOX; or C) DOX+TRZ.** Values were represented as mean ± SD. (*) indicates p < 0.05 as compared to baseline within each group. At baseline, all the groups had n = 20. At the end of 13 weeks, TRZ alone (n = 20); Aliskiren+TRZ (n = 20); Perindopril+TRZ (n = 20); Valsartan+TRZ (n = 20); DOX alone (n = 10); Aliskiren+DOX (n = 16); Perindopril+DOX (n = 14); Valsartan+DOX (n = 14); DOX+TRZ (n = 6); Aliskiren+ DOX+TRZ (n = 14); Perindopril+DOX+TRZ (n = 11); and Valsartan+DOX+TRZ (n = 10).

Echocardiographic indices, including HR, PWT, LVID, LVFS, and LVEF were within normal physiologic limits for all mice at baseline. No significant change from baseline value was observed for HR or PWT at week 13 in any of the treatment arms (Table 1). In C57Bl/6 mice treated with TRZ, there were no significant changes in LV cavity dimensions as compared to baseline (Figure 4A). Mice treated with DOX alone demonstrated a significant increase in cavity dimensions, as the LVID increased from 3.2 ± 0.1 mm at baseline to 4.5 ± 0.2 mm at week 13 (p < 0.05, Figure 4B). Mice receiving DOX+TRZ treatment demonstrated a similar increase in LVID from 3.1 ± 0.2 mm at baseline to 4.6 ± 0.3 mm at week 13 (p < 0.05, Figure 4C). Pre-treatment with Aliskiren, Perindopril, and Valsartan significantly reduced LVID in mice administered DOX alone from 4.5 ± 0.2 mm to 3.6 ± 0.2 mm, 3.9 ± 0.2 mm, and 4.0 ± 0.2 mm, respectively, at week 13 (p < 0.05; Figure 4B). Similarly, in mice treated with DOX+TRZ, the prophylactic administration of Aliskiren, Perindopril, and Valsartan significantly reduced LVID at week 13 from 4.6 ± 0.3 mm to 3.9 ± 0.2 mm, 4.1 ± 0.2 mm, and 4.2 ± 0.1 mm, respectively, (p < 0.05; Figure 4C).

**Table 1:** **Echocardiographic parameters**

**Echo variable**	**Treatment arm**	**Group**	**Baseline**	**Week 13**	**p**
		TRZ	717 ± 12	729 ± 10	0.62
	Placebo	DOX	725 ± 10	701 ± 18	0.58
		DOX+ TRZ	690 ± 14	695 ± 18	0.82
Heart Rate (bpm)		TRZ	735 ± 15	740 ± 12	0.88
	Aliskiren	DOX	705 ± 14	701 ± 21	0.72
		DOX+ TRZ	705 ± 14	701 ± 21	0.72
		TRZ	705 ± 18	710 ± 12	0.9
	Perindopril	DOX	722 ± 14	720 ± 18	0.77
		DOX+ TRZ	722 ± 14	720 ± 18	0.77
		TRZ	744 ± 13	735 ± 12	0.67
	Valsartan	DOX	695 ± 14	705 ± 12	0.72
		DOX+ TRZ	695 ± 14	705 ± 12	0.72
		TRZ	0.82 ± 0.02	0.84 ± 0.01	0.92
	Placebo	DOX	0.83 ± 0.02	0.81 ± 0.01	0.97
		DOX+ TRZ	0.84 ± 0.01	0.83 ± 0.01	0.76
		TRZ	0.80 ± 0.03	0.83 ± 0.02	0.52
	Aliskiren	DOX	0.83 ± 0.02	0.81 ± 0.02	0.68
PWT (mm)		DOX+ TRZ	0.80 ± 0.04	0.82 ± 0.02	0.82
		TRZ	0.81 ± 0.04	0.82 ± 0.02	0.82
	Perindopril	DOX	0.83 ± 0.02	0.81 ± 0.02	0.58
		DOX+ TRZ	0.82 ± 0.04	0.83 ± 0.01	0.76
		TRZ	0.82 ± 0.01	0.84 ± 0.01	0.67
	Valsartan	DOX	0.82 ± 0.03	0.83 ± 0.02	0.72
		DOX+ TRZ	0.84 ± 0.02	0.83 ± 0.01	0.76

PWT, posterior wall thickness.

**Figure 5:**
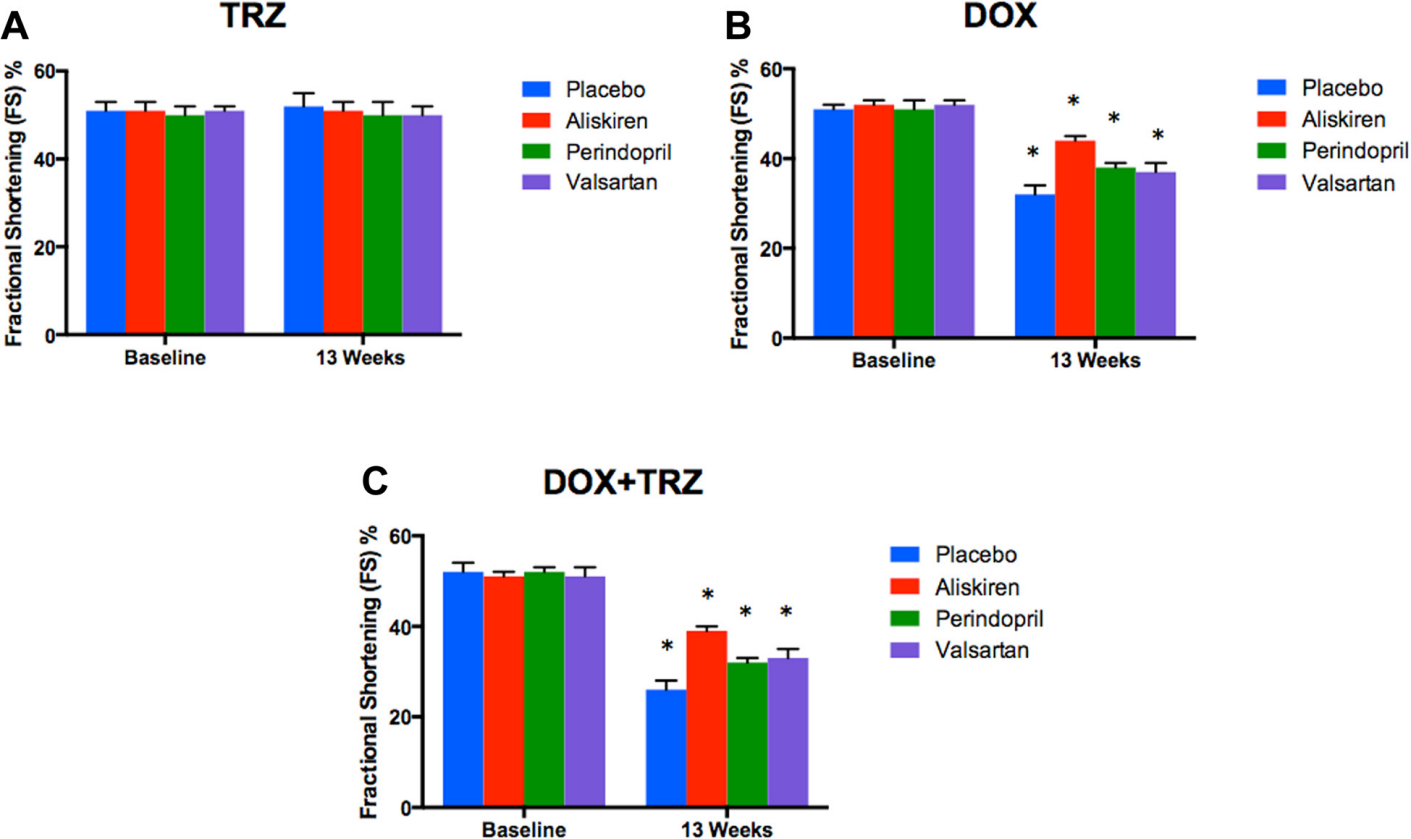
**Fractional shortening of C57Bl/6 mice receiving prophylactic treatment with Aliskiren, Perindopril, Valsartan or placebo prior to receiving one of the following anti-cancer drugs: A) TRZ; B) DOX; or C) DOX+TRZ.** Values were represented as mean ± SD. (*) indicates p < 0.05 as compared to baseline within each group. At baseline, all the groups had n = 20. At the end of 13 weeks, TRZ alone (n = 20); Aliskiren+TRZ (n = 20); Perindopril+TRZ (n = 20); Valsartan+TRZ (n = 20); DOX alone (n = 10); Aliskiren+DOX (n = 16); Perindopril+DOX (n = 14); Valsartan+DOX (n = 14); DOX+TRZ (n = 6); Aliskiren+ DOX+TRZ (n = 14); Perindopril+DOX+TRZ (n = 11); and Valsartan+DOX+TRZ (n = 10).

**Figure 6:**
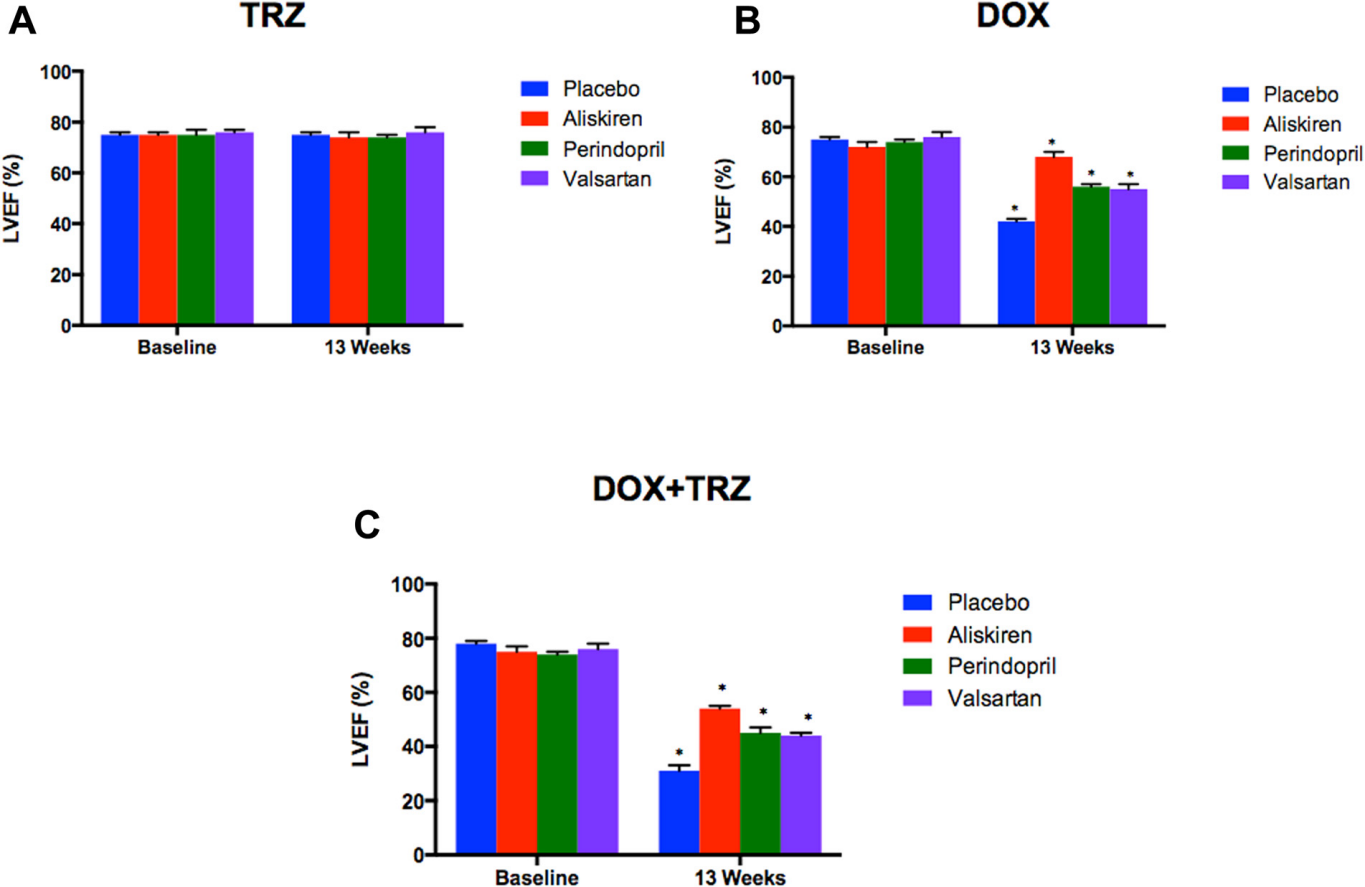
**LV ejection fraction (LVEF) of C57Bl/6 mice receiving prophylactic treatment with Aliskiren, Perindopril, Valsartan or drinking water prior to receiving one of the following anti-cancer drugs: A) TRZ; B) DOX; or C) DOX+TRZ.** Values were represented as mean ± SD. (*) indicates p < 0.05 as compared to baseline within each group. At baseline, all the groups had n = 20. At the end of 13 weeks, TRZ alone (n = 20); Aliskiren+TRZ (n = 20); Perindopril+TRZ (n = 20); Valsartan+TRZ (n = 20); DOX alone (n = 10); Aliskiren+DOX (n = 16); Perindopril+DOX (n = 14); Valsartan+DOX (n = 14); DOX+TRZ (n = 6); Aliskiren+ DOX+TRZ (n = 14); Perindopril+DOX+TRZ (n = 11); and Valsartan+DOX+TRZ (n = 10).

LVFS did not change from baseline values in mice administered TRZ over the course of the 13 week study (Figure 5A). In mice treated with DOX alone, LVFS decreased from 51 ± 1% at baseline to 32 ± 2% at week 13 (p < 0.05; Figure 5B). Pre-treatment with Aliskiren, Perindopril, and Valsartan attenuated DOX induced LV impairment, improving LVFS values to 45 ± 1%, 38 ± 1%, and 37 ± 2%, respectively, at week 13 (p < 0.05; Figure 5B). In mice treated with the combination of DOX+TRZ, LVFS further decreased from 52 ± 2% at baseline to 26 ± 2% at week 13 (p < 0.05; Figure 5C). Prophylactic treatment with Aliskiren, Perindopril, and Valsartan significantly improved LVFS to 40 ± 1%, 32 ± 1%, and 33 ± 2%, respectively, at week 13 (p < 0.05; Figure 5C). The LVEF values corroborated the LVFS findings as shown in Figure 6.

Intra- and inter-observer variabilities of LVID and LVFS parameters are summarized in Table 2. Overall, there was good to excellent intra- and inter-observer variabilities for LVID and LVFS measurements.

**Table 2:** **Intra- and inter-observer variabilities of the echocardiographic parameters**

**Echocardiographic parameters**	**Mean difference ± SD**
**Intra-observer variability**	
LVID (mm)	0.05 ± 0.02
LVFS (%)	0.6 ± 0.2
**Inter-observer variability**	
LVID (mm)	0.08 ± 0.04
LVFS (%)	1.3 ± 0.2

LVID, Left ventricular end diastolic internal diameter; LVFS, Left ventricular fractional shortening.

## Discussion

In Canada, breast cancer is the most common female malignancy and is the second leading cause of cancer related deaths in women. Despite the widespread use of DOX+TRZ in breast cancer treatment, efficacy is limited by the inherent cardiotoxicity of this regimen. Consequently, cardiac dysfunction has been identified in approximately 1 in 4 women receiving this anti-cancer therapy [13-17]. Despite an increased understanding of the potential mechanisms contributing to DOX+TRZ mediated cardiotoxicity, there is currently no approved prophylactic therapy to prevent chemotherapy induced cardiomyopathy in breast cancer patients. In the current study, we demonstrated that the cardiotoxic side effects of DOX+TRZ are partially attenuated by the prophylactic administration of RAS antagonists, as characterized by an improvement in overall survival rate and prevention of adverse cardiovascular remodeling.

Several previous studies using murine models have illustrated the significant mortality associated with DOX administration. Neilan *et al*. demonstrated a survival rate of 35% at week 16 in C57Bl/6 mice receiving DOX (4 mg/kg weekly) for 5 weeks [29]. Similarly, in rats treated with DOX (4 mg/kg weekly) for 6 weeks, a decreased survival rate of 75% was reported at the end of a 10 week study [25]. This present study demonstrated similar reductions in overall survival (50%) for mice in the DOX treatment arm at 13 weeks. The effect was even more pronounced in animals receiving combination therapy with DOX+TRZ. Conversely, prophylactic treatment with RAS inhibitors provided survival benefits. These basic science results provide encouraging evidence for the potential use of RAS antagonists in the prevention of DOX+TRZ mediated cardiomyopathy, which warrants further study in the clinical setting.

In addition to the survival benefits conferred by the prophylactic administration of RAS inhibitors, there is an improvement in adverse cardiovascular remodeling in our chronic DOX+TRZ mediated cardiotoxicity model. Anthracyclines, including DOX, affect hemodynamic and echocardiographic parameters, leading to depressed cardiac function in murine models of chemotherapeutic regimens [26-29]. In an acute murine model of chemotherapy mediated cardiotoxicity, we previously demonstrated severe LV systolic dysfunction due to DOX treatment, as exemplified by LV cavity dilatation and reduced LVEF [26]. The effect was even more pronounced in animals receiving the combination of DOX+TRZ, as the LVFS decreased by 14% and the EF decreased by 20% at day 10, as compared to baseline [26]. Similar results were obtained by Neilan *et al.* in C57Bl/6 mice treated with a single dose of 20 mg/kg DOX [29]. In their study, LVFS and EF decreased by 17 ± 1% and 13 ± 1% respectively, by day 5 [29]. A chronic study of DOX administration (4 mg/kg for 5 weeks) performed by the same group, further demonstrated a reduction in LVFS and EF by 29% and 36%, respectively from baseline value at 16 weeks [29]. Our current study adds to the existing literature by confirming the cardiotoxic effects of DOX in a chronic murine model. Specifically, we demonstrated LV systolic dysfunction, as illustrated by a reduction in LVFS and LVEF and concurrent increase in LVID**.** Furthermore, we demonstrated the increased cardiotoxic side effect in mice administered the combination of DOX+TRZ. In accordance with the aforementioned mortality benefits, RAS inhibition mitigated the adverse cardiac remodelling associated with DOX+TRZ treatment.

Although the pathogenesis of DOX+TRZ mediated cardiotoxicity is multifactorial, there is accumulating evidence to suggest the RAS as a key contributor. Angiotensin II plays an important role as a vasoconstrictor agent and a mitogenic factor by interacting with the AT1_R_ in cardiovascular myocytes [30]. Although DOX induces myofibrillar loss, apoptosis, and significantly impairs contractile function in WT mice, this drug induced cardiotoxicity was not observed in AT1_R_-knockout mice or in animals treated with AT1_R_ blockade [30]. These findings suggest a contributory role for the RAS pathway in the development of DOX mediated cardiotoxicity. The administration of the ARB Telmisartan (10 mg/kg/day for 7 days), was cardioprotective in rats treated with a single dose of 20 mg/kg DOX, by decreasing lipid peroxidation, GSH depletion, and oxidative stress [31]. Additionally, in a chronic rat model of DOX induced cardiotoxicity (DOX 25 mg/kg for a 6 week period), Enalapril served to preserve mitochondrial function, down-regulate free radical production, and preserve overall cardiac function [25]. Finally, in a chronic rat model of DOX (15 mg/kg) mediated cardiac dysfunction, the prophylactic administration of either ACEI (Captopril 60 mg/kg) or ARB (Telmisartan 10 mg/kg) proved to be cardioprotective [32]. This was evidenced by a significant reduction in the concentration of cardiac biomarkers and oxidative stress, and by the maintenance of characteristic cardiac histology in rats pre-treated with RAS antagonism. Our current study adds to the existing literature which supports the cardioprotective capabilities of RAS antagonism in a chronic murine model of chemotherapy induced cardiac dysfunction. Our study is the first to also demonstrate the potential cardioprotective role of direct renin inhibition, specifically Aliskiren, in preventing DOX mediated cardiotoxicity. Additionally, although previous animal studies have focussed on a DOX only model [19,24,25,31,32], we have extended our important findings to a combined DOX+TRZ model, mimicking the clinical setting of breast cancer.

Translating these basic science findings to the clinical arena, a number of clinical studies have evaluated the potential cardioprotective role of RAS antagonists in the clinical setting of chemotherapy mediated cardiotoxicity. Although the administration of ACEI or ARB is associated with reduced morbidity and mortality in the treatment of heart failure [33], a limited number of clinical studies have demonstrated the potential benefits of RAS inhibition in the prevention of DOX mediated cardiotoxicity. In a small prospective study including 49 patients with various cancers responsive to an anthracycline based chemotherapy regimen, Dessi *et al*. demonstrated that ARB was able to reverse acute myocardial dysfunction up to 12 months of follow-up [34]. Cardinale *at al*. demonstrated that ACEI can prevent a decline in LVEF and cardiac events in cancer patients receiving high dose anthracyclines [35]. In a recent meta-analysis, the prophylactic administration of ACEI or ARB in patients receiving an anthracycline based regimen was associated with a relative risk of 0.11 for the development of cardiotoxicity compared to placebo [36]. The recent OVERCOME trial involved 90 patients with malignant hemopathies, randomized to receive prophylactic ACEI (Enalapril) and beta-blockers (Carvedilol) at least 24 hours prior to chemotherapy or to a control group [37]. The results suggested that combination pre-treatment with Enalapril and Carvedilol was cardioprotective, as a lower incidence of death, heart failure and LV systolic dysfunction was observed in the intervention group. The ongoing MANTICORE study is similarly investigating the cardioprotective role of angiotensin converting enzyme inhibition and beta-blockers in HER2 positive breast cancer patients treated with TRZ [38]. Patients were randomized to prophylactic ACEI (Perindopril), beta-blocker (Bisoprolol) or placebo, 7 days prior to TRZ therapy. The MANTICORE study will determine if DOX+TRZ mediated remodelling and systolic dysfunction is attenuated by the prophylactic administration of ACEI and beta-blockers in the clinical setting of breast cancer. In our current study, as the cardiotoxic side effects of DOX+TRZ are partially attenuated by the prophylactic administration of RAS antagonists, in particular by DRI as compared to either ACEI or ARB, future clinical studies evaluating the cardioprotective role of Aliskiren in the breast cancer setting are warranted.

There were some limitations to our study. First, although both DOX+TRZ were co-administered on a weekly basis to produce a chronic murine model of chemotherapy induced cardiotoxicity, adjuvant therapy with TRZ should ideally be given after treatment with an anthracycline based chemotherapy regimen to mimic the clinical setting of breast cancer. Second, although Aliskiren is commonly used as a primary treatment agent for hypertension, recent randomized controlled trials, including the ASTRONAUT study, have failed to demonstrate a benefit of DRI in reducing overall morbidity and mortality in the heart failure setting [39]. In the current study, we demonstrated that Aliskiren may be more cardioprotective as compared to ACEI and ARB, suggesting a need for a clinical study to further evaluate this hypothesis generating finding in the Cardio-Oncology population. Finally, future mechanistic studies focusing on oxidative stress and apoptosis are required to understand the potential cardiprotective effects of the various RAS antagonists in the setting of DOX+TRZ mediated cardiotoxicity.

## Conclusions

The cardiotoxic effects of DOX+TRZ were partially attenuated by the prophylactic administration of RAS antagonists in a chronic murine model of chemotherapy induced cardiac dysfunction.
